# What do patients and informal caregivers value in IBD care? A narrative inquiry

**DOI:** 10.1186/s12913-025-12823-5

**Published:** 2025-05-10

**Authors:** Britt J.M. Thomassen, Evelien M.B. Hendrix, Zlatan Mujagic, Fabiënne G.M. Smeets, Marin J. de Jong, Tom J.G. Gevers, Govert Veldhuijzen, Daniëlle van der Horst, Menne Scherpenzeel, Gerard Dijkstra, Merel L. Kimman, Stephanie M.C. Ament, Marieke J. Pierik

**Affiliations:** 1 Department of Gastroenterology and Hepatology, Maastricht University Medical Centre+, PO box 5800, Maastricht, 6202 AZ The Netherlands; 2 Institute for Nutrition and Translational Research in Metabolism (NUTRIM), Maastricht University, PO box 616, Maastricht, 6200 MD The Netherlands; 3 Department of Surgery, Maastricht University Medical Centre+, PO box 5800, Maastricht, 6202 AZ The Netherlands; 4 Department of Gastroenterology and Hepatology, St. Jans Gasthuis, Vogelsbleek 5, Weert, 6001 BE The Netherlands; 5 Department of Gastroenterology and Hepatology, Horacio Oduber Hospital, Dr. Horacio E. Oduber Hospital Boulevard #1, Oranjestad, Aruba; 6 Department of Gastroenterology and Hepatology, Gelre Hospitals, PO box 9014, Apeldoorn, 7300 DS The Netherlands; 7 Department of Gastroenterology and Hepatology, Radboud University Medical Centre, PO box 9101, Nijmegen, 6500 HB The Netherlands; 8 Crohn & Colitis Netherlands, Houttuinlaan 4b, Woerden, 3447 GM The Netherlands; 9 Department of Gastroenterology and Hepatology, University Medical Centre Groningen, University of Groningen, PO box 30.001, Groningen, 9700 RB The Netherlands; 10 Department of Clinical Epidemiology and Medical Technology Assessment (KEMTA), Maastricht University Medical Centre+, PO box 5800, Maastricht, 6202 AZ The Netherlands; 11 Care and Public Health Research Institute (CAPHRI), Maastricht University, PO box 616, Maastricht, 6200 MD The Netherlands; 12 Department of Innovation - Centre of Expertise Quality, Innovation & Research, Maastricht University Medical Centre+, PO box 5800, Maastricht, 6202 AZ The Netherlands

**Keywords:** Inflammatory bowel disease, Patient values, Person-centred care, Qualitative research, Informal caregivers

## Abstract

**Background:**

Inflammatory Bowel Disease (IBD) is an increasingly prevalent chronic condition that impacts the lives of patients and their relatives, and burdens healthcare systems. Redesigning care processes is warranted, with digital technologies offering opportunities to increase efficiency and reduce workloads. However, successful innovations require meaningful involvement of patients and informal caregivers. In this study, we aimed to identify what IBD patients and their informal caregivers value in IBD care.

**Methods:**

A purposive sample of 18 IBD patients and 8 informal caregivers was drawn from an academic and a non-academic hospital in the Netherlands. Semi-structured interviews with a narrative approach focused on capturing real-life experiences and personal stories related to IBD care. Transcripts were analysed using inductive thematic analysis.

**Results:**

Patients and informal caregivers valued clear information and support regarding the disease, treatment, and daily management, along with active involvement in treatment planning. Regular contact with the same trusted healthcare professionals (HCPs), and effective treatment that alleviates symptoms and enables normal living, were deemed important. Patients appreciated a holistic, personalized approach. They welcomed remote care for follow-up and self-management, as long as the remote monitoring program was trustworthy and included easily accessible outpatient care if needed. Informal caregivers valued attentive HCPs who take patients seriously.

**Conclusions:**

Key elements of IBD care valued by patients and informal caregivers include comprehensive support, active involvement, a person-centred approach, easy access to outpatient care, regular follow-ups with trusted HCPs, and remote care complementing outpatient services. Innovation teams should consider these elements when refining and developing innovations in IBD care.

**Supplementary Information:**

The online version contains supplementary material available at 10.1186/s12913-025-12823-5.

## Background

Inflammatory bowel disease (IBD) is a chronic condition characterized by recurring inflammation of the gastrointestinal mucosa, affecting approximately five million individuals globally, including 120,000 in the Netherlands in 2019 [1, 2]. The disease course of IBD is heterogeneous, complex and often progresses in the long term, leading to a reduced quality of life for patients [3]. IBD significantly impacts not only patients, but also their family, friends and social circle [4]. Close relatives and friends often act as informal caregivers, a role that can result in considerable distress and burden [5, 6].

Due to a rising incidence of IBD and increasing overall life expectancy of IBD patients, without proportional growth in the number of healthcare professionals (HCPs), healthcare services tend to become overburdened [7]. This, along with a shift in disease management from conventional and surgical therapies to novel, often expensive, treatment options, has contributed to increased healthcare costs [8, 9]. To ensure that high-quality care remains accessible and affordable for the growing population, transformation in the organization and delivery of IBD care is pivotal [10].

Digital health technologies, such as remote monitoring platforms, show promise in supporting innovative care processes. Other healthcare innovations, including point-of-care testing and redesigned care pathways, are gradually being integrated into routine care as well [11]. These innovations empower patients by giving them more responsibility and control over their care, while easing pressure on the healthcare system through more effective use of limited staff resources [12].

For healthcare innovations to be valuable, implementable and sustainable, active participation of patients and their informal caregivers in the innovation process is crucial [13, 14]. Their lived experiences provide unique insights that complement those of HCPs [15], helping to identify key elements of care, preferences, and unmet needs. Previous studies show that IBD patients prioritize e.g., shared decision-making, treatments that relieve symptoms, and flexible follow-up care [3, 16–18]. Additionally, family members of IBD patients benefit from psychosocial support to improve family function, and enhanced coping strategies [19]. While patient and informal caregiver involvement in developing innovations is widely recognized, existing studies rarely use narrative methodology to capture core values based on lived experiences, and the informal caregiver perspective remains underrepresented. Furthermore, these studies frequently overlook the organizational, innovative, and future-oriented aspects of IBD care. Addressing this gap is particularly important in the Dutch context, where ongoing changes in IBD care delivery and the rapid advancement of innovations require a comprehensive understanding of patient and informal caregiver perspectives. Moreover, the European Crohn’s and Colitis Organisation (ECCO) advocates for context-specific criteria to define quality of care, which have not yet been established for the Netherlands [20].

Therefore, the aim of this study was to gain a comprehensive understanding of what patients and their informal caregivers value in IBD care. These insights can guide the development and implementation of innovations, particularly in areas such as remote monitoring using eHealth tools, and the reorganization of IBD care. Additionally, these key elements of IBD care can support the refinement of existing innovations or care processes, aligning them with the needs, preferences and values of end users.

## Methods

A qualitative study with a narrative approach was conducted to gather stories and real-life experiences from patients with IBD and their informal caregivers. In semi-structured interviews, participants were asked to share their experiences of living with IBD, their interactions with IBD-related healthcare, and their views on what elements of IBD care are of value to them. This qualitative study is reported in accordance with the Consolidated Criteria for Reporting Qualitative Research (COREQ) [21].

### Setting

In the Netherlands, IBD care is primarily managed by a core team of hospital-based HCPs comprising a gastroenterologist, nurses specialized in IBD or nurse practitioners, supported by a multidisciplinary team of, for example, surgeons, dieticians, and psychologists. Remote care pathways and digital health technologies, such as the telemonitoring platform myIBDcoach and point-of-care faecal calprotectin tests, have been gradually implemented and integrated into Dutch IBD care and are now used by many hospitals in the Netherlands [11].

### Study participants and sampling

Patients diagnosed with IBD and their informal caregivers were recruited from outpatient clinics at an academic hospital with a remote monitoring care pathway (Maastricht University Medical Centre+, MUMC+) and a non-academic hospital without a remote monitoring care pathway (St. Jans Gasthuis Weert) in the Netherlands. Participants aged 18 years or over who were sufficiently acquainted with the Dutch written and spoken language were eligible for inclusion. A convenience sampling method was employed; however, participant characteristics were monitored during recruitment to ensure diversity in age, gender, educational level (i.e., low: primary education, pre-vocational secondary education, lower years of senior general secondary education or pre-university education; intermediate: upper years of general secondary education or pre-university education, or vocational education; high: higher professional education or university education) [22], ethnic background, disease phenotype (i.e., Crohn’s disease or ulcerative colitis), disease duration, perceived disease activity, and experience with telemonitoring in IBD care. Eligible patients received an information sheet and an informed consent form from their IBD HCP. The researcher (BT) then contacted patients by phone to provide information, clarify questions, and emphasize that participation was voluntary. Participants were reimbursed for any travel expenses, and informed that the interview would last approximately 30 to 60 min. Patients who agreed to participate were asked to invite an informal caregiver, such as a family member (i.e., partner, parent, child, sibling) or a close friend, to take part in the study. Informal caregivers could choose to join the patient in a dual interview, or both could participate in an individual interview, depending on their preference. Recruitment continued until data saturation was reached in both groups, meaning that no new information emerged from the interviews [23].

### Data collection

Separate interview guides for patients with IBD and informal caregivers were developed by BT and SA, consisting of semi-structured, open-ended questions (Table 1, see Supplementary file 1 for the full interview guides) that encouraged participants to share their stories and experiences in depth. Two pilot interviews, one with a patient and one with an informal caregiver, were conducted to assess the clarity and relevance of the questions. Based on these interviews, a question regarding the variety of healthcare professionals involved in patient care was added to the interview guide.

**Table 1:** Main topics from the interview guides for patients and informal caregivers

Daily life & IBD	• IBD disease course • Impact of IBD on daily life • Management of IBD
Received care	• Type of care received by IBD patient • Experiences with IBD care • Involvement of informal caregivers in IBD care
High quality IBD care	• Elements of high quality IBD care • Experiences with high quality and poor IBD care • Changes and improvements in IBD care • Experiences and expectations related to eHealth and telemonitoring • Practical aspects of IBD care

Participants were recruited from March to December 2023. The interviews took place at the hospital, via Microsoft Teams, or by phone, depending on the participants’ preference. Each participant was interviewed once, and all interviews were audio recorded. Directly following each interview, notable findings and relevant nonverbal observations were saved as field notes. Recordings were transcribed verbatim via Amberscript software and verified by the researcher (BT). Participants received a summary of their interview as a member check and were invited to confirm the interpretation of the findings or provide additional comments, which were also documented [23]. All documents were pseudonymized via codes (P[number] for patients and IC[number] for informal caregivers) and securely stored within the protected environment of the MUMC+.

The first author (BT), a PhD candidate trained in qualitative research, conducted the interviews under the guidance of an experienced qualitative researcher (SA). None of the researchers (BT, EH, or SA) had prior relationships with the participants or were involved in their care.

### Data analysis

Given the narrative and explorative nature of the interviews, an inductive thematic analysis approach was employed to identify key aspects and elements of IBD care that are valuable for patients and informal caregivers. The interviews, including the pilot interviews, with patients and informal caregivers were analysed separately for each group. For dual interviews involving both a patient and an informal caregiver, only the parts where the patient contributed were included in the patient analysis, and similarly, only the informal caregiver’s contributions were included in the caregiver analysis. Three researchers (BT, EH and SA) were involved in the coding and analysis process to ensure triangulation. Two researchers (BT, and EH or SA) independently coded the first seven patient and four informal caregiver transcripts, prioritizing those with the most extensive information. The research team then convened to compare codes and reach consensus in case of differences in interpretation. Preliminary themes and subthemes were identified, leading to the development of draft coding trees. After open coding revealed that the Picker Principles of person-centred care could help to structure the emerging themes, these principles were used to provide an initial categorization of the codes to obtain a feeling with the data [24]. These coding trees were applied to four additional patient transcripts and two informal caregiver transcripts, with new and adapted codes incorporated if needed. The final seven patient transcripts and two informal caregiver transcripts were coded by one researcher using the updated coding trees. Finally, analysis of the pilot interviews using the coding trees revealed no new topics, confirming data saturation was reached [23]. NVivo software (version 14) was used to manage the coding process and develop coding trees.

## Results

Eighteen patients and eight informal caregivers participated in an interview. Participant characteristics are shown in Table 2. Five patients participated in a dual interview with their informal caregivers, while thirteen patients and three informal caregivers participated in individual interviews. Eight interviews were conducted in person, seven by phone, and six via video call. The interviews had a mean duration of 43 min (range 23–74 min).

**Table 2:** Characteristics of IBD patients and informal caregivers

	Number of participants, n (%)	
	IBD patients (*n* = 18)	Informal caregivers (*n* = 8)
Age		
18– 30 years	2 (11.1)	1 (12.5)
31– 45 years	4 (22.2)	1 (12.5)
46– 60 years	4 (22.2)	1 (12.5)
61– 75 years	7 (38.9)	5 (62.5)
> 75 years	1 (5.6)	0
Gender		
Female	10 (55.6)	5 (62.5)
Male	8 (44.4)	3 (37.5)
Education level		
Low	4 (22.2)	0
Intermediate	7 (38.9)	5 (62.5)
High	7 (38.9)	3 (37.5)
Ethnic background		
Dutch	18 (100.0)	7 (87.5)
Other; born in Africa, raised in Europe	0	1 (12.5)
Relation with patient		
Spouse	NA	4 (50.0)
Parent	NA	3 (37.5)
Sibling	NA	1 (12.5)
Hospital*		
Academic hospital	10 (55.6)	6 (75.0)
Non-academic hospital	8 (44.4)	2 (25.0)
Disease phenotype*		
Crohn’s disease	9 (50.0)	6 (75.0)
Ulcerative colitis	8 (44.4)	1 (12.5)
IBD-unclassified	1 (5.6)	1 (12.5)
Time since official diagnosis*		
< 2 years	3 (16.7)	2 (25.0)
2– 5 years	3 (16.7)	1 (12.5)
6– 10 years	3 (16.7)	4 (50.0)
11– 25 years	4 (22.2)	1 (12.5)
> 25 years	5 (27.8)	0
Perceived disease activity*		
Remission	9 (50.0)	3 (37.5)
Mild complaints	6 (33.3)	3 (37.5)
Active disease	3 (16.7)	2 (25.0)
Experience with telemedicine*, yes	10 (55.6)	6 (75.0)

*IBD* Inflammatory Bowel Disease, *NA* not applicable

*characteristics of informal caregivers related to those of their relatives with IBD

**Fig. 1:**
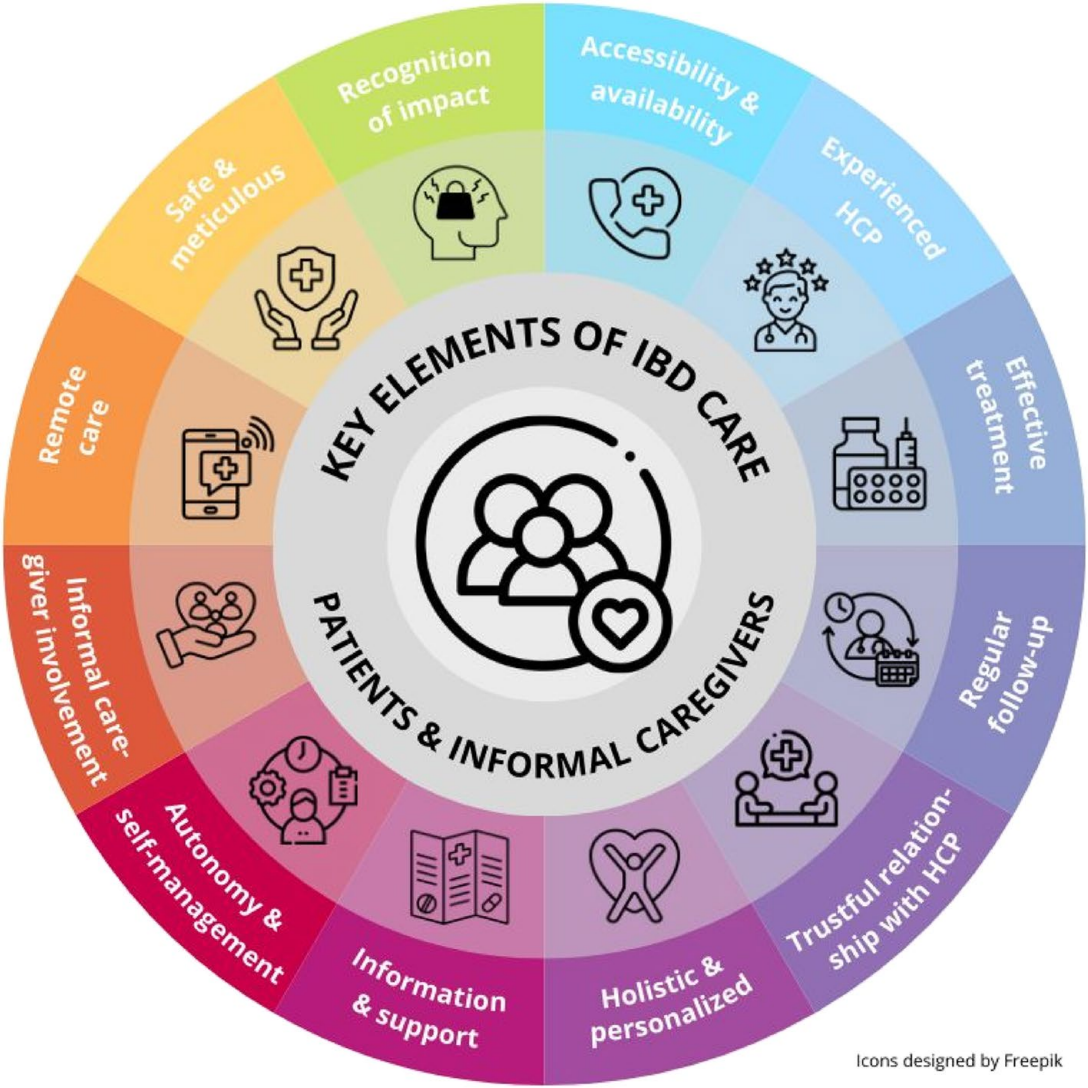
Key elements of IBD care valued by patients with IBD and their informal caregivers

The main findings of the interviews, i.e., the key elements of IBD care valued by patients and informal caregivers, are shown in Fig. 1 and are discussed in further detail below.

### IBD care is accessible and available

All patients and informal caregivers stressed the importance of being able to ask questions easily and receiving prompt, specific responses from IBD-specialized HCPs. Quick access to their HCPs was considered vital for addressing medical concerns; patients valued the ability to visit the hospital directly or on short notice in urgent situations.

[regarding IBD care with a telemonitoring tool] “Because there are no barriers; if you have a question, you can just ask it, or if you feel something in the evening, you can ask that question right away, and you’ll know by the next morning. So yes, the accessibility is low-threshold, and it’s easy.” (IC03)

Patients who felt experienced in assessing their own medical needs expect immediate action from the hospital with minimal waiting times when they reach out for help.

“I have never experienced being kept on hold; if there is truly something going on, I can get an appointment the very next day.” (P05)

It was deemed important for patients to have easy access to individuals who can assist them in scheduling appointments without experiencing long hold times. Appointments were sometimes arranged without prior consultation, which can hinder patients’ ability to incorporate them into their daily lives, including work and other commitments.

Furthermore, high-quality IBD care must be accessible nearby. Some patients were concerned that healthcare reorganization would lead to centralized specialized care in only a few hospitals in the country, requiring longer travel times. Those treated at multiple hospitals reported variations in organizational structure and quality of care. They underscored the need for uniformity in IBD treatment across hospitals. Patients and informal caregivers acknowledged the strain on healthcare services and understood that solutions are not easily attainable, accepting the current situation.

### HCPs are specialized and experienced in IBD

Patients expected their HCPs to be specialized in IBD, knowledgeable about their patient’s medical history and records, and experienced in managing the condition.

“I sometimes hear that they want to move IBD care to the general practitioner, with a practice nurse specifically for IBD patients… I’m not sure if they would have enough expertise.” (P17)

Both patients and informal caregivers valued referrals to other IBD specialists or HCPs from different specialties when needed for their specific expertise.

“A doctor who says they will refer you, and six months later, it still hasn’t happened. That was a bad experience.” (P11)

Some informal caregivers highlighted the importance of timely referrals from general practitioners to hospitals for patients with IBD-like symptoms, noting that earlier diagnosis could have alleviated significant suffering. Participants appreciated HCPs who strived to diagnose medical complaints promptly and identify optimal treatment options effectively, while also being supportive and addressing practical issues in a pragmatic manner. Overall, clear communication among patients, informal caregivers and HCPs within the interdisciplinary setting was deemed essential; patients valued effective collaboration among HCPs from different specialties. To achieve this, one informal caregiver emphasized the need for high-quality medical education.

“In surgery, they don’t seem to know that I have Crohn’s.” (P04)

### Effective treatment that reduces IBD-symptoms and minimizes impact on people’s lives

All patients highlighted the importance of effective treatment to minimize IBD symptoms, allowing them to lead a normal life.

“It never truly goes away, but what I hope for is to not feel so sick anymore. I hope they have the right medication for me or something that will allow me to live well again and grow old.” (P15)

Participants expressed hope for future curative medications, but they also raised concerns about the potential side effects of current treatments. Access to comprehensive information regarding these side effects and treatment consequences was considered essential. Patients shared practical experiences with both intravenous treatments and self-administered injections, valuing options that minimized disruption to their daily lives. Costs were also a concern, with patients and informal caregivers recognizing the high expenses of medications and expressing a desire to reduce waste. Overall, while satisfied with the ordering and administration process regarding medication, some patients showed interest in exploring alternative treatment options. Many patients sought a clear overview of available treatment options and predictive tools to identify effective therapies, as they often had to try multiple treatment options before finding one that worked. Some patients have participated in clinical trials, emphasizing the need for ongoing research due to the absence of a cure.

### Regular follow-up conducted with the same HCP

Participants highly valued regular follow-ups and monitoring by HCPs of the hospital, including scheduled appointments with their HCP, as well as routine blood and faecal tests. While these tests were not difficult or burdensome, some patients found stool sample collection inconvenient, particularly because of the unpredictability of bowel movements or the need to collect samples at work.

Maintaining regular contact with the same HCP was crucial for patients and informal caregivers, as it minimized the need to repeatedly explain the patient’s medical history, and helped build trust. Patient preferences for follow-up frequency varied; some preferred contact only when medical issues or questions arose, whereas others valued regular consultations regardless of their health status.

“That you can return annually and aren’t left on your own, that you are supported as you grow older with this.” (P15)

“I think the most important thing is to feel heard, to be familiar, so that you don’t have to tell at every visit again who you are, what your problem is, when the last time was that you had a problem or issues, not having to repeat yourself constantly.” (P02)

### A familiar and trusted HCP who has sufficient time and attention for you as a person

Patients and informal caregivers emphasized the importance of a trusting relationship with their HCP, which is built through regular face-to-face appointments and HCPs honouring their commitments. Participants valued a friendly, attentive, and respectful approach by reception staff and HCPs, highlighting the importance of being listened to and taken seriously. They stressed that HCPs should dedicate sufficient time and attention to understand patients’ IBD-related issues and address them effectively, which helps reduce symptoms and disease burden. This approach allows patients to feel seen as individuals rather than just as a number within the broader healthcare system. Informal caregivers emphasized the importance of patients feeling that HCPs take them seriously.

“You are not treated as a number there [in the hospital]. It feels like she [nurse specialist] is your friend.” (IC04)

“What is important is that you don't feel brushed off. We understand very well that not everything can be solved, but the feeling of being dismissed, like,'Oh, here they come again,' seems terrible to me. Fortunately, I have never had that feeling, and I don't think my son has either." (IC02)

During the transition from paediatric to adult IBD care, a patient and informal caregiver noted the reduced monitoring and support in the adult system, with the caregiver expressing a preference for more frequent consultations with both care teams.

### HCPs provide care using a holistic and personalized approach

Overall, patients and their informal caregivers valued HCPs who recognize them as individuals and adopt a holistic perspective, considering not only the medical aspects of their condition, but also other aspects of life affected by the disease, such as mental health and social life.

“It’s very focused on the disease, and maybe a bit more attention could be given to the person behind it. So, zooming in on your living situation, and they might be able to offer more tips or tricks to improve your quality of life.” (P12)

One patient appreciated her HCPs support in exploring alternative medicine alongside regular treatment. Others noted a link between stress and IBD symptoms and expressed a desire for guidance on stress management in daily life. Providing targeted, personalized answers to personal questions was deemed important, as it helps alleviate concerns and fears.

“Start psychological support earlier. Take into account that there are also waiting times, which can lead to more psychological issues than if the support had been initiated sooner.” (P06)

### Relevant, clear and comprehensive information and support on the disease, treatment, and daily management of IBD

Patients and informal caregivers valued sufficient, relevant, clear and comprehensive information, support and guidance regarding their disease, treatment options and daily living with IBD, including diet and lifestyle advice. This need for guidance and support was especially important after diagnosis, when patients often felt overwhelmed in processing all available information.

“Then that doctor called back afterward to say: ‘yes, [name], you’re going to get a higher dose of medication because it’s necessary; the others aren’t working well anymore, and these work better. So we’ll see how we can proceed with this medication.’ So far, it’s going reasonably well. I think it’s really wonderful that a doctor called my daughter to explain.” (IC08)

Patients appreciated when HCPs directed them to practical resources, such as a restroom access card or support groups. Some patients also connected with others via the Dutch IBD patient association (Crohn & Colitis NL) to discuss living with IBD. All this support and information helped patients manage their condition proactively.

“The hospital handed me several tools, for example a medical toilet pass. Things you wouldn’t come up with yourself. They also told me about this group with other patients, where you can ask each other questions and help each other. I think that’s good.” (P05)

Furthermore, patients and informal caregivers expressed a desire to raise awareness about the impact of IBD, noting that friends, colleagues and the general public often lack an understanding of how it affects daily life.

“Maybe that friends and family can also receive a folder or pamphlet about how you can support someone who is dealing with IBD? What can you expect? What can you do when they feel ill? You know, those kind of things. Nobody knows anything about it.” (P15)

### Patients are involved in their care and have autonomy to manage their condition

Patients emphasized the importance of collaboration with their HCPs and valued active involvement in making their treatment plans, which included sharing their opinions and participating in decision-making. Patients and informal caregivers expected all treatment options to be discussed, with clear explanations of the treatment sequence, and alternatives if the current medication would fail. Patients also desired autonomy in managing their care, such as preparing discussion points for consultations, and felt responsible for tracking their health status.

“You truly notice that your wishes are being listened to and that your well-being is considered important, more important than the potential effects of infusions on the long- or short-term. You feel heard, and it reinforces the idea that expressing your feelings is worthwhile. It shows that your opinion truly matters.” (P01)

A few patients mentioned that they felt persuaded by their HCP to switch to a cheaper biosimilar, leading to adverse side effects and poor outcomes. They viewed this as inadequate care, feeling that their preferences were ignored and that side effects were not properly addressed. Additionally, patients appreciated clear explanations from HCPs during procedures such as endoscopy, even if they did not fully grasp everything due to sedation.

### Informal caregivers are informed about the treatment plan and receive guidance on supporting individuals with IBD

Informal caregivers were generally not directly involved in the medical management of IBD. Their role was mainly supportive, especially outside the clinical setting, accompanying patients to hospital visits. Other tasks of informal caregivers included monitoring the patient’s condition, encouraging rest, intervening when needed, providing emotional support, and engaging in enjoyable activities to aid the patient’s well-being.

“Very good support, and that’s very important from the doctors and from us as parents, that we are there for her. If something comes up, we talk about it, which alleviates a lot.” (IC08)

Most informal caregivers did not perceive living with a relative who has IBD as burdensome; however, they expressed concerns about their relative’s health, particularly when patients did not communicate their discomfort. They emphasized the need for accurate and honest updates during hospital admissions, especially when their relative’s condition is critical or changes quickly. Furthermore, informal caregivers sought guidance on supporting individuals with IBD, assisting with practical aspects of chronic illness (e.g. acquiring materials for disease management, addressing financial concerns), and valued opportunities to connect with other caregivers to share experiences.

### Remote IBD care is available for patients

Most patients favoured remote care when the disease was stable, finding hospital visits unnecessary and time-consuming. However, others preferred face-to-face appointments because they were unfamiliar with remote care and valued personal interaction. Importantly, all respondents emphasized that remote care should never replace face-to-face care completely. Opinions on video consultation varied; some patients felt it lacked a personal touch, whereas others found it sufficiently interactive since they could still see their HCP. Most agreed that sharing laboratory results or making simple medical decisions could be efficiently managed over the phone.

According to participants, the benefits of remote care included easy access to ask questions, receiving quick responses, monitoring health from home (although some patients reported issues with the applications), ordering medication, and accessing reliable information on topics such as lifestyle and mental health.

“I like that it [telemonitoring tool] can be digital, it makes it easier and allows you to incorporate it more easily into your daily routine.” (IC03)– “It makes healthcare more accessible.” (P03)

Most patients preferred to manage IBD-related tasks at home when possible and to visit the hospital only when necessary. Monitoring disease activity via faecal calprotectin was appreciated, as it reduced the need for frequent hospital visits to hand in stool samples. However, some patients found the tests impractical in use.

Patients highlighted the flexibility of telemedicine, enabling them to engage with the healthcare system at their convenience. Some found the questionnaires, patient-reported outcome measures (PROMs), used for health assessment beneficial for reflection, whereas others deemed them too lengthy, frequent, or irrelevant. Concerns were raised about losing contact with the hospital, as it was unclear who reviewed their responses and when, along with a lack of clarity regarding upcoming medical check-ups.

“I definitely prefer face-to-face contact over online interactions, submitting things, and receiving a questionnaire from someone I don’t know. I’ve always felt this way; I appreciate personal connection more. I’d rather go to the doctor than fill out a questionnaire and wonder who’s reviewing it, who’s judging it, and who makes decisions based on it?” (P02)

### IBD care process is perceived as safe and meticulous

One patient raised concerns about telemedicine, feeling a lack of control, as the hospital might miss issues if patients do not complete questionnaires or inaccurately report their condition as stable. Another patient mentioned that despite reporting problems in the questionnaire, no action was taken by HCPs, leading this patient to switch to more traditional methods, such as phone or e-mail, and stop using the remote system. Some patients felt the need to closely monitor their IBD care due to errors, such as appointment scheduling issues or miscommunication between departments, contributing to feelings of anxiety and concern.

“I’ve noticed that, for example at the pharmacy department, they don’t seem to know that I’m on Infliximab. I think they should know that to assess whether certain medications can be used together. I find that a little concerning at times.” (P04)

In addition, some patients sometimes had to reach out to the hospital for follow-up appointments, making them worried that they had been forgotten by the IBD care team. For informal caregivers, it was also important that patients feel closely monitored by HCPs.

“You need to keep a close eye on things yourself; otherwise, things will go wrong.” (P14)

### The burden and impact of IBD on patients are fully recognized

The impact and burden of IBD varied among patients and their informal caregivers. While some patients faced significant discomfort, others in long-term remission reported fewer issues. Many patients noted that living with IBD became part of their routine, a sentiment echoed by informal caregivers. Common challenges reported by patients included chronic fatigue, dietary restrictions, difficulties resuming pre-diagnosis work, and the impact of IBD on their sex lives. Patients often felt embarrassed due to urgency for restroom use or faecal incontinence, which led them to avoid activities such as vacations and theatre visits.

“Then you’re 39, and you’re on a long flight with a diaper on. Psychologically, that does have an effect on you.” (P06)

They also expressed discomfort with hospital admissions, particularly when they shared a bathroom with others.

“Once I stayed in the hospital, I had to go to the toilet 40 times a day and couldn’t hold it at all. When you’re in a room with five patients and have to share the bathroom with so many others, that just doesn’t work.” (P11)

Besides, IBD was considered time-consuming; patients spent considerable time attending hospital appointments, sometimes undergoing regular infusions or performing daily transrectal irrigation for faecal incontinence, which needs to be coordinated with their work schedules. In addition, patients expressed frustration that friends, family, and their HCPs often fail to understand the profound impact of the condition on their lives.

## Discussion

This qualitative, narrative study examined what patients and informal caregivers value in IBD care. Key elements included comprehensive information and support for disease management, treatment options, and daily living with IBD. Patients emphasized the importance of care that fosters safety, autonomy, and self-management, along with effective treatments that reduce symptoms and support daily functioning. Both patients and informal caregivers also highlighted the need for easy access to communication with HCPs to address questions and acute issues, regular follow-ups with trusted HCPs, and a holistic, personalized approach where patients feel like they are being taken seriously. While remote IBD care was valued, it should complement rather than replace outpatient services.

The elements identified by IBD patients and their informal caregivers align with general patient values described by Bastemeijer et al., who include categories of patients (uniqueness and autonomy), professionals (compassion, professionalism and responsiveness), and interactions (partnership and empowerment) [25]. They also correspond to the Picker Principles of person-centred care, such as access to care, respect for patients’ preferences, coordination and integration of care, physical comfort, emotional support, involvement of family and friends, information and education, and continuity of care and smooth transitions [24]. These findings suggest broader generalizability to other patient populations.

Our findings underscore a strong patient need for information and support, closely linked to the desire for autonomy and effective self-management of IBD. Maintaining a normal life was a critical priority for patients in our study, a theme also highlighted in a meta-synthesis of qualitative research on living with IBD, where patients emphasized striving for a normal life while managing their condition [3]. Autonomy support from HCPs can further enhance self-management in patients with IBD, especially during disease relapse [26]. Focusing on self-care, psychological coping, disease-specific health literacy, self-evaluation and social interaction skills can enhance the empowerment of IBD patients [27]. Health literacy plays a critical role, enabling patients to understand health information and apply it to decision-making, disease management, and communication with HCPs. Therefore, it is essential that information about IBD for patients and informal caregivers is easy to understand [28].

Many elements mentioned in our study relate to communication and relationships between patients, informal caregivers, and their HCPs. Patient-physician communication and the quality of their relationship significantly influence health outcomes, treatment adherence, trust, and patient satisfaction with treatment decisions [29–31]. Patients valued HCPs who viewed them as individuals, addressing not only the medical aspects of IBD but also its effects on mental health, social life, and overall well-being. This underscores the importance of a holistic, person-centred approach, which is increasingly emphasized in advancing IBD care, with a focus on adopting multidisciplinary care and integrating value-based care aligned with patients’ definitions of high-quality care [32].

Another main theme in our study, and broadly in healthcare innovation, is the use of digital health and remote care pathways, such as telemonitoring, home-based faecal calprotectin testing via digital applications, and video consultations. Both patients in our study and those in previous research acknowledge the benefits of these innovations, such as personalized care, easy access to HCPs, remote monitoring of disease and health, and access to reliable information, while also addressing the growing burden on IBD care [11, 33, 34]. However, our findings reveal that while patients and informal caregivers are willing to engage in remote care during remission, they still value easy access to outpatient care for medical issues and prefer regular in-person appointments with their HCPs. Some participants noted reduced quality of care due to limited face-to-face interaction with their HCP, a finding that is consistent with previous research [35]. These mixed perspectives highlight the need to tailor care to patient preferences to enhance their overall experience of care [18, 36].

The elements and needs expressed by informal caregivers in our study, such as the need for information about the disease and treatment plan and guidance to support their loved ones, align with findings from other studies [37, 38]. A systematic review highlighted how IBD impacts family members’ emotional well-being, relationships, social lives, work, finances, and leisure activities, as well as their need for support groups and psychosocial counselling [19]. However, informal caregivers in our study did not view their relative’s IBD as burdensome. This could be due to adaptive coping strategies, e.g., acceptance, resilience, or positive reframing [19]. Moreover, it could also be reluctant to express personal challenges during dual interviews with their relative with IBD. Differences in healthcare system organization, financing, or cultural factors may also explain this discrepancy, as reliance on informal caregivers differs across countries [37]. In clinical practice, HCPs should recognize and address the impact of IBD on family members and ensure that they are informed and actively involved in the treatment and care of their relative with IBD.

Overall, other studies have explored the needs and values of IBD patients regarding treatment across diverse contexts and methodologies, yielding findings that are consistent with our study [17, 18, 36, 39–41]. However, in light of rapidly evolving care pathways and innovations, particularly in digital health, our study provides up-to-date insights specific to patients and their informal caregivers within the Dutch healthcare context.

### Strengths and limitations

The strengths of this study primarily lie in its narrative approach, which provides an in-depth understanding of what individuals with IBD and their informal caregivers value in IBD care. This methodology facilitated the exploration of real-life experiences and emotions, highlighting participants’ voices and perspectives on their needs and preferences. The narrative approach also allowed for a comprehensive discussion of all aspects of IBD and care that were important to patients and informal caregivers. Researcher triangulation and member checks further strengthened the methodological rigor [23]. A diverse group of patients and informal caregivers from a large academic hospital known for its digital healthcare innovations, as well as a smaller, more traditional hospital participated, providing valuable insights across different healthcare settings. Additionally, the mean age of the patient sample (52.7 years) was similar to that of the overall Dutch IBD population, which ranges from 50.9 to 54.9 years [2]. However, only one participant had an ethnic background other than Dutch, which may limit the generalizability of our findings to patients and informal caregivers from different cultural backgrounds. Another limitation to consider is that some interviews were conducted by telephone, restricting the observation of nonverbal communication. However, participants were given the option to choose their preferred interview method (telephone, video call, or in-person) to maximize their comfort.

### Future research

The results of this study can inform the development of quality of care standards, as current recommendations often rely primarily on HCP expert opinions rather than on the values of patients and informal caregivers [15, 20, 42]. It is also important to consider the perspectives of other stakeholders involved in IBD care, such as healthcare providers, health insurers, pharmaceutical companies, and eHealth providers. To innovate sustainably and successfully, it is essential to account for the elements of IBD care valued by all stakeholders. The insights gained from such studies can be used to guide high-quality and sustainable innovation, while improving IBD care broadly. Emerging innovations in this field include for example, further development of telemonitoring tools, the use of eNurses for remote patient support, video consultations, home-administered medications, and wearable sensors that measure inflammatory markers in real time [43]. Implementing these innovations requires changes in care processes at an organizational level. The insights from this study can serve as core values to maintain during these transitions of care.

## Conclusions

This qualitative study highlights that IBD patients and informal caregivers value comprehensive support for disease management, autonomy, effective treatments, and a personalized, holistic approach where they feel heard. Key elements include easy access to outpatient care, regular follow-ups with trusted HCPs, and the use of remote care to complement outpatient services. These insights can guide future innovations in IBD care, ensuring alignment with the needs and preferences of IBD patients and their informal caregivers.

## Supplementary Information

Supplementary Material 1.

## Data Availability

The datasets used and/or analysed within this study are available from the corresponding author on reasonable request.

## Abbreviations

COREQ: Consolidated Criteria for Reporting Qualitative Research
HCP: Healthcare professional
IBD: Inflammatory Bowel Disease
IC: Informal caregiver
MUMC+: Maastricht University Medical Centre+
NL: The Netherlands
PROMs: Patient-reported outcomes measures
