# Supplementary material for: What do patients and informal caregivers value in IBD care? A narrative inquiry
Source: BMC Health Serv Res. 2025 May 10;25:681. doi: 10.1186/s12913-025-12823-5 (PMC12065271; doi:10.1186/s12913-025-12823-5)
Supplement: Supplementary file 1 — Supplementary Material 1. [file 12913_2025_12823_MOESM1_ESM.docx]

**Supplementary file 1. Interview guides of patients and informal caregivers**

| **Patients** | **Informal caregivers** |
| --- | --- |
| **Daily life & IBD** | |
| How are you doing? | |
|  | How long has your relative had IBD, and how is he/she doing? |
| What do you do in daily life? | |
| What does your illness mean to you? | What does your relative’s illness mean to you? |
| To what extent does your illness hinder you in daily life? | To what extent does the illness hinder you and your relative in daily life? |
| How do you manage your illness? |  |
| **Received care** | |
| What type of care do you receive? | What type of care does your relative receive? |
| Who provides this care? | |
| How do you perceive it? | |
| Who is involved with your care? | Who are you involved with as an informal caregiver? |
| How is that going? | |
| **Good IBD care** | |
| What is important for good IBD care, and why? | What is important for good IBD care for your relative, and why? |
|  | What is important in your role as an informal caregiver? |
| What is going well in the current care? | |
| What could be improved? | |
| Can you share an experience of good or poor IBD care? | |
| How would you define good IBD care? | |
| How has care changed since your diagnosis? | How has care changed since your relative’s diagnosis? |
| What has changed? | |
| What is missing in current IBD care? | |
| What is important for home monitoring? | |
| How is the process for faecal and blood tests? |  |
| What are your wishes for future IBD care if everything were possible? | |
| What is the most important aspect of good IBD care? | |
| **Ending** | |
| Is there anything you would like to mention that was not covered? | |
